# The impact of leaflet resection versus leaflet preservation on left ventricular reverse remodelling after mitral valve repair: insights from the UK mini mitral trial

**DOI:** 10.1186/s13019-026-04060-6

**Published:** 2026-06-09

**Authors:** Eteesha Rao, Christopher D. Bayliss, Janelle M. Wagnild, Richard Graham, Rebecca Maier, Enoch Akowuah

**Affiliations:** 1 Newcastle University Medical School, Newcastle Upon Tyne, UK; 2 Department of Cardiac Surgery, James Cook University Hospital, South Tees Hospitals NHS Foundation Trust, Middlesbrough, UK; 3 Department of Anthropology, Durham University, South Road, Durham, UK; 4 Academic Cardiovascular Unit, James Cook University Hospital, South Tees Hospitals NHS Foundation Trust, Middlesborough, UK; 5 Population Health Sciences Institute, Newcastle University, Newcastle upon Tyne, UK; 6 Translational and Clinical Research Institute, Newcastle University, Newcastle upon Tyne, UK

**Keywords:** Mitral valve repair, Chordal implantation, Leaflet preservation, Leaflet resection, Reverse remodelling, Resect, Respect

## Abstract

**Objectives:**

Mitral valve repair (MVr) promotes left ventricular (LV) reverse remodelling, and subsequent reduction in LV diameters and volumes. The effectiveness of different MVr techniques, *Resect* (leaflet resection) and *Respect* (leaflet preservation) remains debated. This study aimed to investigate the impact of *‘Resect’* and ‘*Respect’* techniques on LV reverse remodelling, primarily assessed by changes in indexed LV end-systolic diameter (ILVESD) post-MVr.

**Methods:**

Data were drawn from the UK Mini Mitral Trial, which randomised 330 patients to MVr via mini-thoracotomy or median-sternotomy. This subanalysis included patients with isolated posterior leaflet prolapse, who had undergone MVr via *Resect* or *Respect* techniques. Transthoracic echocardiography was performed pre-operatively and at 12 and 52 weeks post-operatively, with all studies analysed by a core laboratory. Linear mixed-effects models compared changes in indexed LV measurements between groups.

**Results:**

This subanalysis included 175 patients (36 *Resect* and 139 *Respect)*. Annuloplasty was performed in all but one repair. We found no significant difference between groups in recurrent MR severity at 52 weeks postoperatively (*p* = 0.31). Both groups had significant improvement in ILVESD by 52 weeks (mean change from baseline − 5.6%, *p* < 0.001), with no difference between groups (*p* = 0.24). Both techniques led to significant improvements in indexed end-diastolic LV volumes and diameters, decreasing from baseline to 12 and 52 weeks. However, LV end-systolic volumes and diameters increased at 12 weeks before decreasing by 52 weeks postoperatively.

**Conclusion:**

No significant differences in LV reverse remodelling between *Resect* and *Respect* techniques. Both can be recommended to surgeons. Longer follow-up is needed.

**Graphical Abstract:**

**Figure:**
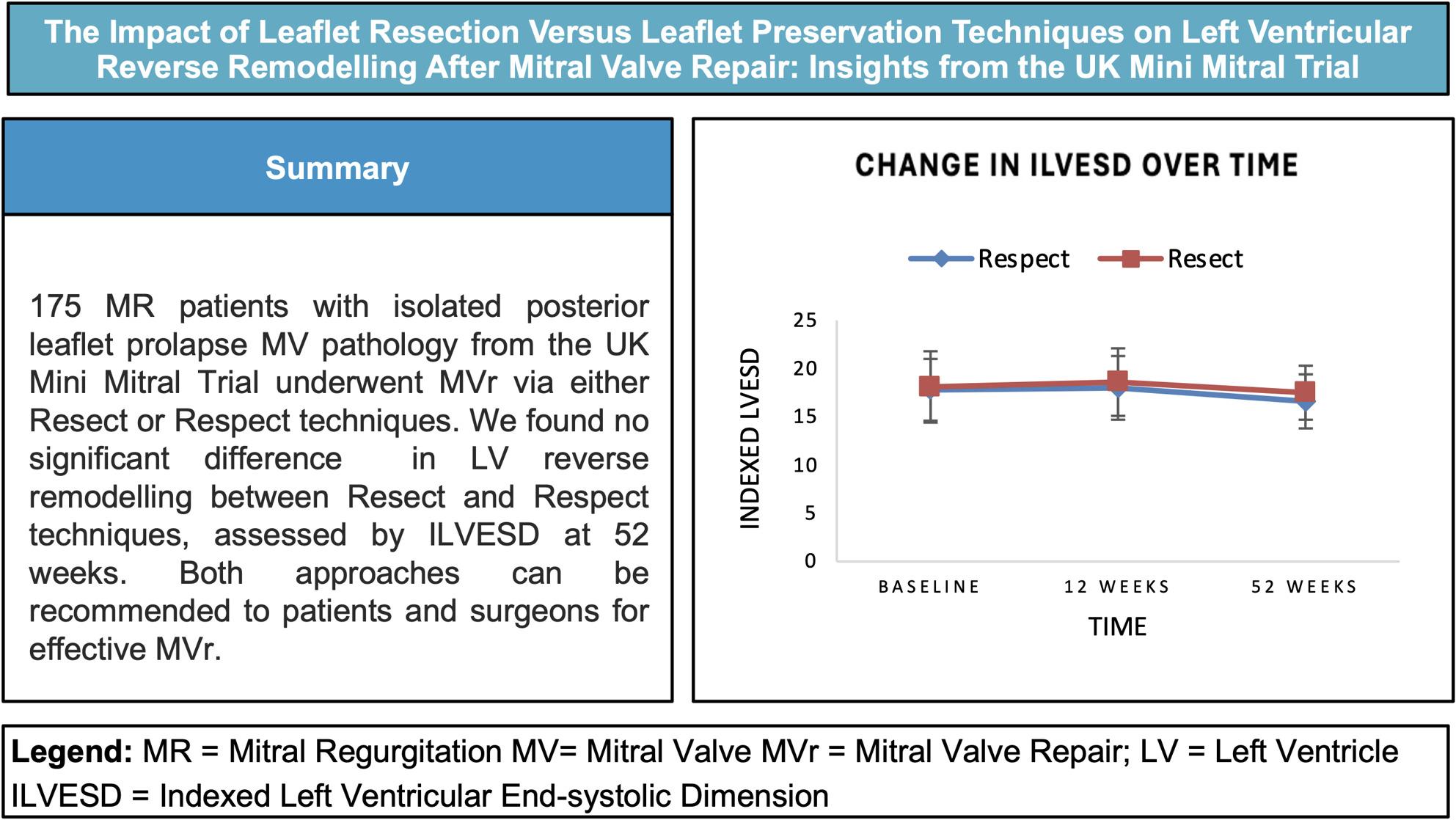

## Introduction

Mitral valve repair (MVr) is the preferred surgical approach for treating patients with severe mitral regurgitation (MR) [1] Globally, MR is the second most common valvular abnormality with a prevalence of 2–10%, which increases with age [2–4]. Severe MR can reduce normal life expectancy without intervention, with an average mortality rate of ~ 5% per year and 70% at 8 years [2].

In chronic progressive MR, left ventricular (LV) remodelling occurs to compensate and allow for the cardiac output to be maintained. Initially, there may be an observed increase in LV ejection fraction (LVEF), however the effective ejection fraction may be reduced, dependent on the volume of the regurgitant fraction [2, 3]. As the disease progresses, a positive feedback loop may establish due to the volume overload, causing LV dilatation and eccentric remodelling [2, 3]. After patients with MR undergo MVr, the LV normally undergoes reverse remodelling, reverting towards its normal morphology over time. The exact sequence for these changes has been debated with studies reporting different patterns.

Some studies, such as Shafii et al. 2012 have reported a significant decline in LV end-systolic diameters (LVESD) and LV end-diastolic diameters (LVEDD) at one year following MVr, whilst Suri et al., 2009 found that only the LVEDD had a significant decline with LVESD not changing appreciably postoperatively until three to five years [5, 6]. Similar discrepancies exist regarding postoperative changes in LV volumes, with Le Tourneau’s two-stage phased model being the preferred hypothesis [7, 8]. The two-stage phased model proposed by Le Tourneau et al. (2019), based on their single centre retrospective study, compared non-indexed LV dimensions and volumes after MVr during the early postoperative period (within 45 days of MVr surgery) with the late postoperative period (mean 428 days). In the first phase, during the immediate postoperative period, they found a reduction in LVEDV with no change in left ventricular end-systolic volume (LVESV). This was followed by a second stage of reverse LV remodelling, featuring a decrease in LVEDV and LVESV [8].

In most western populations, posterior leaflet mitral valve (MV) prolapse due to degenerative MV disease remains the most common MV pathology. This can be attributed to the ageing population. Posterior leaflet MV pathologies were traditionally repaired using the ‘*Resect’* approach, first described and evidenced in 1983, as “the French correction” by Alain Carpentier [9]. This approach involves resecting away the leaflet tissue and became the gold standard technique until more recent wider adoption of the *‘Respect’* approach, which has increasingly been reported to be an equally effective, if not arguably superior approach [10–12]. The *Respect* technique involves the placement of one or more expanded polytetrafluoroethylene neo-chords, without resecting any leaflet tissue. This was later proposed as an alternative approach that may allow for preserved leaflet mobility with minimal changes to annular geometry and the ability to implant larger annuloplasty rings [13, 14]. Thus, creating the recent shift in MVr surgical practice to ‘*Respect rather than Resect’* [10]*.*

Over the past three decades, several studies have compared *Resect* and *Respect* MVr techniques using various outcomes, commonly: residual MR severity, recurrent MR, reoperation rates and LV changes [15]. Some studies have found no significant difference between *Resect* and *Respect* in the aforementioned outcomes [7, 15–18]. Others found *Resect* to be superior [19–20], whilst some found *Respect* to outperform *Resect *[2, 10–12, 21, 22].

A major limitation with the current studies is that they predominantly have a small sample size and/or are single centre observational studies, with the potential to be influenced by selection bias [17, 20, 21, 23]. Thus, it is difficult to definitively state whether one repair technique is superior to the other. To our knowledge, there are currently only three published studies assessing the impact of MVr technique on LV reverse remodelling: two single centre observational studies and one small randomised controlled trial (RCT) [7, 23, 24].

This study aims to use high-quality data from the UK Mini Mitral Trial to address previously reported discrepancies in the literature and ascertain if there are any differences in LV reverse remodelling between *Resect* and *Respect* approaches at 52 weeks post-MVr.

## Patients and methods

### Ethical statement

The UK Mini Mitral Trial was granted ethical approval by the NHS Wales Ethics Committee 6 (16/WA/0156). This sub-analysis is covered by the same ethical approval.

### Study population: the UK mini mitral trial

The UK Mini Mitral Trial was a multicentre, superiority RCT which randomised 330 patients with degenerative MR between November 2016 and January 2021. The trial protocol has been previously published [25, 26].

This subanalysis only included patients with isolated posterior leaflet pathology that had undergone MVr through *Resect* or *Respect* approaches. Patients were excluded if they had: anterior or bileaflet pathologies, received MV replacement, any other MVr techniques (excluding ring annuloplasty) or not completed the 52-week echocardiography core laboratory follow-up.

### Surgical techniques

MVr was carried out through mini-thoracotomy or conventional median-sternotomy. Patients undergoing MVr via the *Resect approach* had the prolapsing segment of the posterior leaflet excised via either triangular or quadrangular resection (+/- sliding plasty). All patients undergoing the *Respect* approach underwent chordal reconstruction using artificial expanded polytetrafluoroethylene (ePTFE). A ring annuloplasty was also performed for all but one repair. Concomitant procedures for atrial fibrillation or tricuspid valve repair were allowed in the trial. Patients requiring other additional procedures or emergency surgery were excluded. All operations were performed by experienced cardiac surgeons who had performed at least 50 MVr surgeries previously.

### Outcomes

The primary outcome of this study was the difference in indexed LVESD, measured by transthoracic echocardiography (TTE) assessed by a core laboratory, compared between *Resect* and *Respect* groups from baseline to 52 weeks after MVr surgery.

Secondary outcomes were early differences in indexed LV end-systolic and end-diastolic measurements from baseline to 12 weeks postoperatively; late differences in indexed LV end-systolic and end-diastolic measurements from baseline to 52 weeks postoperatively and differences in severity of residual MR at 52 weeks postoperatively between *Resect* and *Respect* groups.

### Echocardiography

All patients underwent TTE at three specified time points. These were at baseline (prior to surgery), early post-operative period (within 12 weeks after surgery) and late post-operative period (52 weeks after surgery). A central blinded independent core laboratory adjudicated echocardiographic data from all patients at all three time points to obtain data on LV diameters and volumes at end-systole and end-diastole.

### Statistical analysis

Statistical analyses were conducted using IBM SPSS version 29.0.2.0 [27]. Comparisons of categorical variables were conducted using chi-squared or Fisher’s exact test, depending on sample size. Continuous variables were assessed using independent t-tests if normally distributed or the Mann-Whitney U test if non-normally distributed.

Primary and secondary outcomes were analysed using linear mixed-effects models adjusted for surgical approach (mini-thoracotomy versus sternotomy) and the variables used as minimisation factors in the UK-Mini’s randomisation scheme (presence of AF (present/absent), severity of tricuspid regurgitation (none/mild/moderate/unknown), and the patient’s baseline Physical Function Score based on the SF-36v2 (low (0–33), moderate (> 33–66), high (> 66)). Intra-patient correlation was taken into account using a random intercept model. Time was included as a fixed effect to estimate changes in indexed LV diameters and volumes from baseline to 52 weeks post-MVr, regardless of *Resect* or *Respect* technique. To determine whether changes in indices over time differed by MVr technique, tests for interaction between time and technique were included in the model. Statistical significance was set at *p* < 0.05 with mean differences and 95% confidence intervals reported.

## Results

**Fig. 1:**
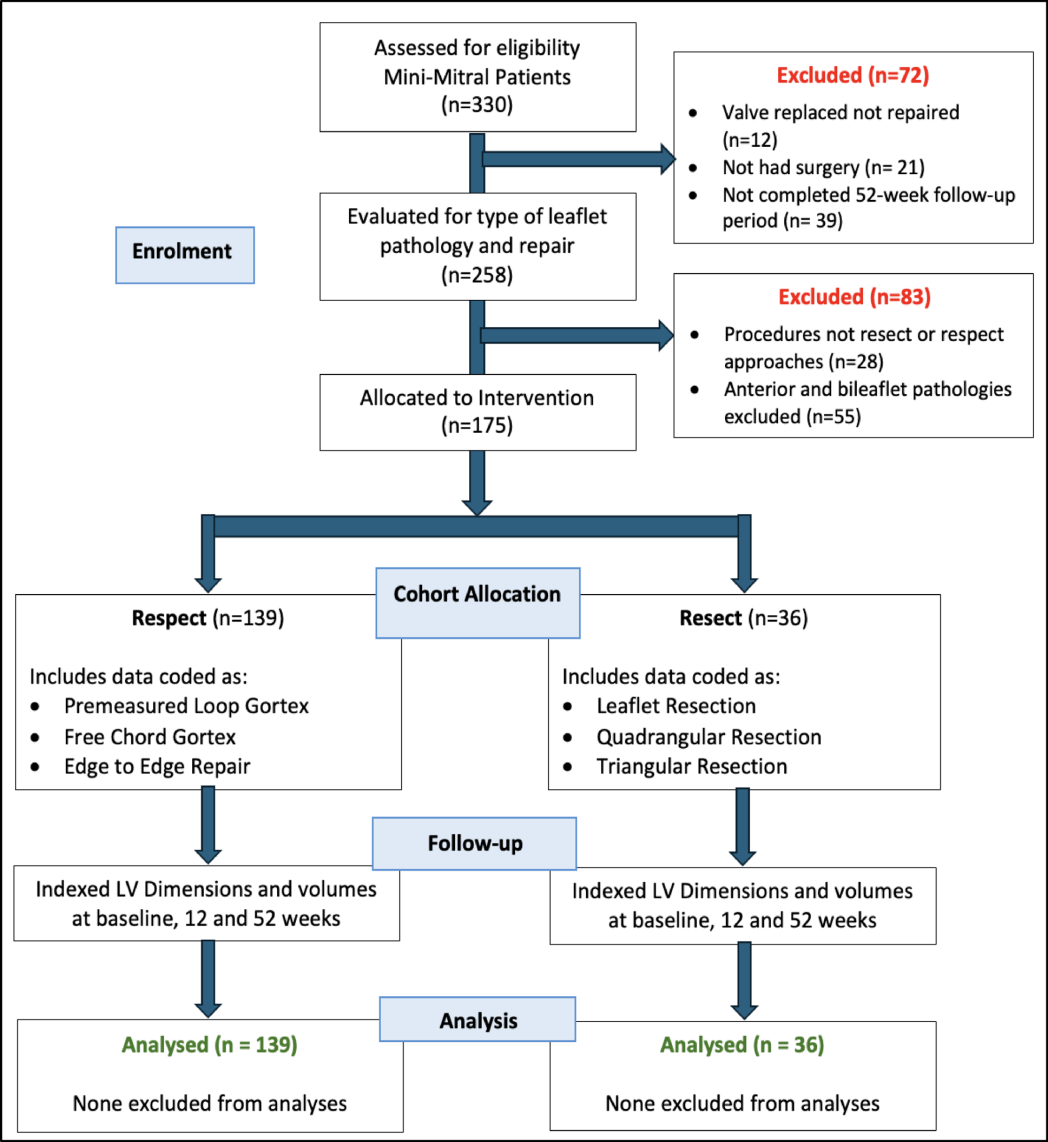
CONSORT diagram

After assessing the data from all 330 patients in the UK-Mini, 175 patients met the inclusion criteria with 36 *Resect* and 139 *Respect* patients for the comparative analyses, refer CONSORT diagram in Fig. 1.

### Patient demographics

Baseline patient demographics were compared between *Resect* and *Respect* groups and are displayed in Table 1. Patients in the *Respect* group were more likely to have a higher NYHA classification than those in the *Resect* group.

**Table 1:** Baseline patient characteristics

		Total *n* = 175	Respect *n* = 139	Resect *n* = 36
**Age, years, Mean ± SD**		66 ± 10	66 ± 10	66 ± 10
**Male**, n (%)		131 (75)	103 (74)	28 (78)
**NYHA class**, n (%)				
1		23 (13)	19 (14)	4 (11)
2		59 (34)	39 (28)	20 (56)
3		70 (40)	62 (45)	8 (22)
4		23 (13)	19 (14)	4 (11)
**NYHA class > 2**, n (%)		93 (53)	81 (58)	12 (33)
**BMI**, mean ±SD		26.4 ± 4.2	26.5 ± 4.0	25.9 ± 4.7
**EuroSCORE II**, Mean ± SD	*n* = 174	1.58 ± 1.3	1.62 ± 1.3	1.42 ± 1.2
**Clinical History**, n (%)				
Atrial fibrillation		65 (37)	53 (38)	12 (33)
Tricuspid Regurgitation	*n* = 35	24 (69)	17 (12)	7 (19)
Heart failure		51 (29)	41 (29)	10 (28)
Diabetes		13 (7)	10 (7)	3 (8)
Pulmonary hypertension		32 (18)	27 (19)	5 (14)
Previous myocardial infarction		4 (2)	4 (3)	0 (0)
Stroke		9 (5)	6 (4)	3 (8)
Chronic obstructive pulmonary disease		9 (5)	8 (6)	1 (3)
Asthma		12 (7)	8 (6)	4 (11)
Renal failure		6 (3)	6 (4)	0 (0)
Peripheral vascular disease		1 (<1)	0 (0)	1 (3)
**Urgency**, n (%)				
Elective		153 (87)	121 (87)	32 (89)
Urgent		22 (13)	18 (13)	4 (11)
**Carpentier class**, n (%)				
Type I		8 (5)	7 (5)	1 (3)
Type II		155 (89)	121 (87)	34 (94)
Type I + II		12 (7)	11 (8)	1 (3)
**SF-36v2-PF**, n (%)				
Low (<33)		37 (21)	31 (22)	6 (17)
Moderate (33-66)		61 (35)	49 (35)	12 (33)
High (>66)		77 (44)	59 (42)	18 (50)

### Intraoperative details

Intraoperative details were compared between *Resect* and *Respect* groups, as summarised in Table 2. Triangular (67%) and quadrangular (33%) resection were the two *Resect* techniques used. All *Respect* patients underwent chordal reconstruction with artificial chordae.

**Table 2:** Intraoperative details

		Total population *n* = 175	Respect group *n* = 139	Resect group *n* = 36	Risk ratio (respect vs. resect: 95% CI)	*p* value
**Approach, ***n* (%)						<0.001
Minimally invasive		89 (51)	80 (58)	9 (25)	1.31 (1.12, 1.54)	
Sternotomy		86 (49)	59 (42)	27 (75)		
**MVr technique**, n (%)						
Leaflet resection						
Triangular resection			0 (0)	24 (67)		
Quadrangular resection			0 (0)	12 (33)		
Neochord reconstruction			139 (100)	0 (0)		
**Ring annuloplasty**, n (%)		174 (99)	139 (100)	35 (97)		
Complete ring		150 (86)	122 (88)	28 (80)		
Incomplete ring		24 (14)	17 (12)	7 (20)		
					**Mean difference (respect-resect) [95% CI]**	
**Ring size**,** mm** Mean ± SD	*n*= 174	32 ± 2.5	31 ± 2.3	33 ± 2.5	-2.24 [-3.12,-1.36]	<0.001
**Perfusion details** Mean ± SD						
Cardiopulmonary bypass time, min		114 ± 34	117 ± 33	101 ± 36	15.9 [3.62, 28.2]	0.01
Aortic cross-clamp time, min		82 ± 74	84 ± 83	73 ± 20	11.5 [-16.0, 38.9]	0.41

**Fig. 2:**
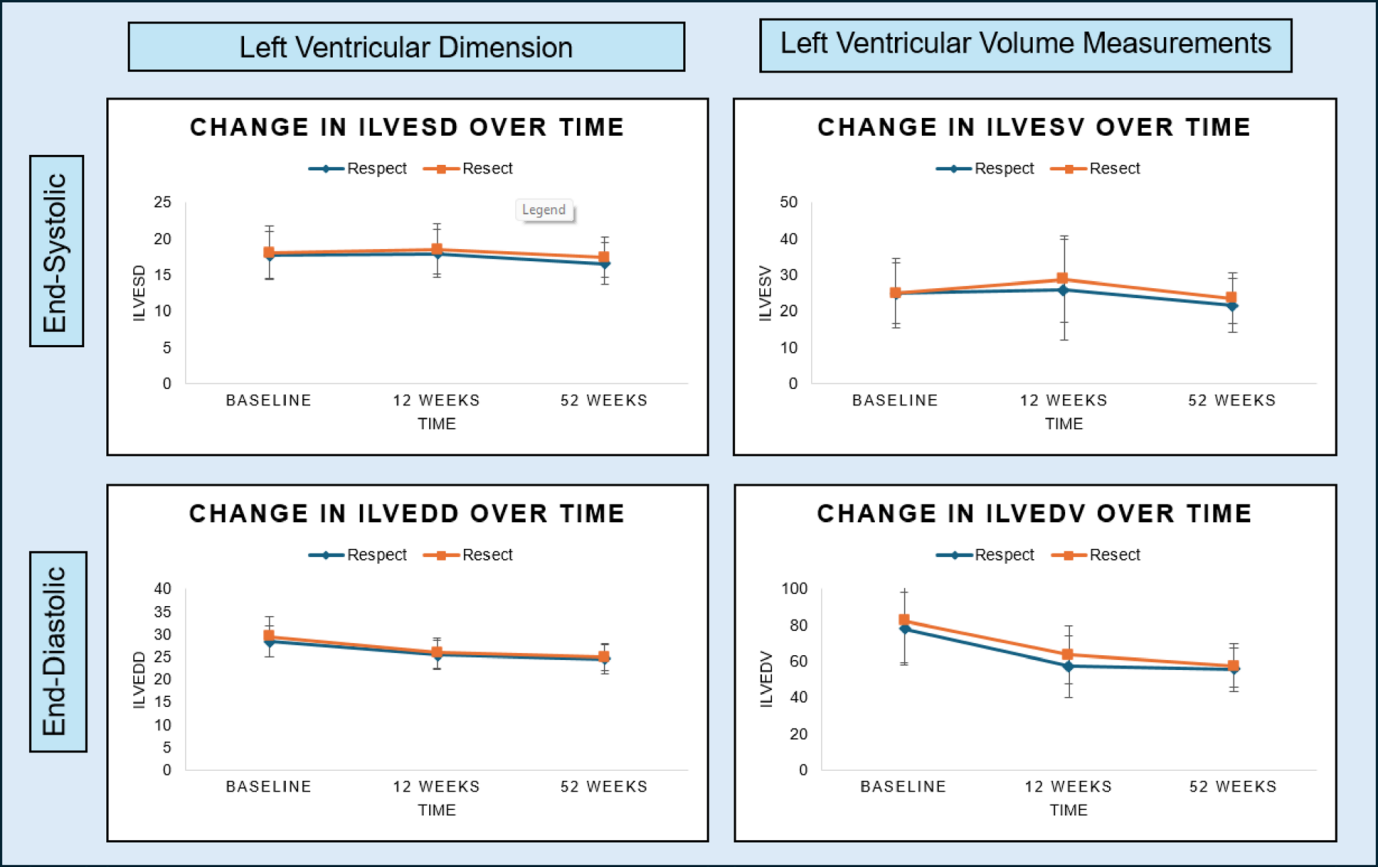
Left ventricular changes from baseline to 52 weeks: *Resect* vs. *Respect* MVr techniques

The surgical approach used for the MVr was significantly different (*p* < 0.001) between groups, with the *Respect* technique being the preferred option in patients undergoing the minimally invasive approach (89.9% *Respect* versus 10.1% *Resect*). The *Respect* patients also had longer bypass times (*p* = 0.01), likely due to the higher uptake of the minimally invasive approach with the *Respect* technique. All patients except one in the *Resect* group received an annuloplasty ring. There was a significant difference in annuloplasty ring type and size between groups (*Resect* group mean 33 mm versus *Respect* group mean 31 mm, *p* < 0.001), refer Fig. 2.

### MR severity at 52 weeks: resect versus respect

We observed no significant difference in qualitative MR severity between *Resect* and *Respect* groups at 52 weeks postoperatively (*p* = 0.31).

### Primary outcome: results

We found no significant difference in change in ILVESD (mm/m²), measured by TTE, between *Resect* and *Respect* groups from baseline to 52 weeks (*p* = 0.24), refer Fig. 2.

### LV remodelling post-MVr: total population

Across the total patient population in this study (*n* = 175), there was a significant reduction in ILVEDD and ILVEDV post-MVr regardless of repair technique at 12 weeks post-MVr as compared to baseline (see Table 3). There was a significant increase (*p* = 0.03) in ILVESV and a non-significant increase in ILVESD (*p* = 0.18) in this early postoperative period. However, by the late postoperative period, at 52 weeks, there was a significant decrease in all indexed LV end-systolic and end-diastolic volumes and diameters post-MVr regardless of repair technique as compared to baseline.

**Table 3:** Comparison of indexed LV diameters and volumes from baseline to 12 and 52 weeks

				Total Patients (*n*=175)			
	Baseline	12 weeks	52 weeks	Baseline to 12 weeks difference		Baseline to 52 weeks difference	
	Mean ± SD	Mean ± SD	Mean ± SD	Mean difference (12 weeks-baseline: [95% CI])	*p* value	Mean difference (52 weeks-baseline: [95% CI])	*p* value
ILVEDD mm/m^2^	28.7 ± 3.7 (*n*=171)	25.6 ± 3.2 (*n*=170)	24.6 ± 3.2 (*n*=175)	-3.04 [-3.47, -2.60] (*n*=166)	<0.001	-3.99 [-4.46, -3.53] (*n*=171)	<0.001
ILVESD, mm/m^2^	17.9 ± 3.3 (*n*=171)	18.2 ± 3.4 (*n*=170)	16.8 ± 2.8 (*n*=175)	0.31 [-0.15, 0.76] (*n*=166)	0.18	-1.01 [-1.53, -0.61] (*n*=171)	<0.001
ILVEDV, mL/m^3^	79.0 ± 21 (*n*=170)	58.7 ± 17 (*n*=170)	56.0 ± 12 (*n*=171)	-20.1 [-22.7, -17.5] (*n*=166)	<0.001	-23.0 [-26.1, -20.0] (*n*=166)	<0.001
ILVESV, mL/m^3^	25.0 ± 9.4 (*n*=170)	26.6 ± 13 (*n*=170)	22.1 ± 7.3 (*n*=171)	1.62 [0.14, 3.09] (*n*=166)	0.03	-2.95 [-4.23, -1.6] (*n*=166)	<0.001

### Early LV changes post-MVr: resect versus respect

In the early postoperative period at 12 weeks, there was no significant difference in ILVESD (*p* = 0.35), ILVEDD (*p* = 0.56), ILVEDV (*p* = 0.06) and ILVESV (*p* = 0.24) post-MVr between *Resect* and *Respect* techniques (refer Table 4). There was also no significant difference in mean transmitral gradient (*p* = 0.34) and coaptation length (*p* = 0.25) between groups.

**Table 4:** Post-MVr echocardiographic findings from baseline to2 and 52 weeks with differences between*Resect* and *Respect* groups

	Respect group (*n*=139)			Resect group (*n*=36)			Difference at baseline	Resect vs. Respect: difference of changes			
	Baseline	12 weeks	52 weeks	Baseline	12 weeks	52 weeks		Baseline to 12 weeks		Baseline to 52 weeks	
	Mean ± SD	Mean ± SD	Mean ± SD	Mean ± SD	Mean ± SD	Mean ± SD	*p* value [95% CI]	Mean difference (respect-resect: [95% CI])	*p* value	Mean difference (respect-resect: [95% CI])	*p* value
ILVEDD, mm/m^2^	28.5 ± 3.4 (*n*=136)	25.5 ± 3.2 (*n*=135)	24.5 ± 3.3 (*n*=139)	29.5 ± 4.4 (*n*=35)	25.9 ± 3.3 (*n*=35)	25.0 ± 3.0 (*n*=36)	0.13 [-2.42, 0.32]	0.42 [-0.66,1.50]	0.45	0.60 [-0.55, 1.76]	0.31
ILVESD, mm/m^2^	17.8 ± 3.2 (*n*=136)	18.0 ± 3.3 (*n*=135)	16.6 ± 2.8 (*n*=139)	18.1 ± 3.7 (*n*=35)	18.6 ± 3.5 (*n*=35)	17.5 ± 2.8 (*n*=36)	0.64 [-1.54,0.95]	-0.49 [-1.60,0.63]	0.39	-0.69 [-1.82,0.45]	0.24
ILVEDV, mL/m^3^	78.2 ± 20 (*n*=137)	57.5 ± 17 (*n*=135)	55.6 ± 12 (*n*=136)	82.2 ± 23 (*n*=33)	63.5 ± 16 (*n*=35)	57.6 ± 12 (*n*=35)	0.32 [-12.0, 3.94]	-1.77 [-8.28, 4.85]	0.60	2.15 [-5.46,9.94]	0.58
ILVESV, mL/m^3^	25.0 ± 9.6 (*n*=137)	26.0 ± 14 (*n*=135)	21.7 ± 7.4 (*n*=136)	25.1 ± 8.4 (*n*=33)	28.9 ± 12 (*n*=35)	23.6 ± 6.9 (*n*=35)	0.96 [-3.70, 3.52]	-2.59 [-6.27,1.10]	0.17	-1.62 [-4.85,1.62]	0.33

### Late LV changes post-MVr: resect versus respect

In the late postoperative period at 52 weeks, there was no significant difference in ILVEDD (*p* = 0.31), ILVEDV (*p* = 0.57) and ILVESV (*p* = 0.33) between *Resect* and *Respect* techniques as compared to baseline, refer Table 4.

## Discussion

This observational, multicentre, cohort study assessed postoperative outcomes for 175 MR patients with degenerative posterior leaflet pathology, who had undergone MVr via either the *Resect* or *Respect* technique. The main finding of this study was that there is no significant difference in LV reverse remodelling between *Resect* and *Respect* approaches at 52 weeks post-MVr surgery.

Our findings are in line with the only published RCT but not in keeping with previous single centre, observational studies by [7, 23, 24]conducted the only RCT comparing *Resect* and *Respect* MVr techniques and their effect on LV reverse remodelling. 104 patients from seven Canadian centres were included. 54 patients were randomised to the Resect group and 50 patients to the Respect group. They measured indexed LV end-systolic and end-diastolic measurements in the early postoperative period and at one year, finding no significant difference in LV reverse remodelling between techniques. The time period for their early postoperative period was immediately before discharge and did not match with our study, which was within 12 weeks of MVr surgery [23].

The propensity-matched retrospective study Del Forno et al. (2023) matched 170 patients to *Resect* and *Respect* groups in a 1:1 ratio. In contrast to our results, they found that the *Respect* group had improved reverse LV remodelling as compared to the *Resect* group [24]. They reported a higher reduction in LVEDD in the *Respect* group at last follow-up (*p* < 0.001) with average last follow-up being at 5.97 years (4.49–9.61 years). It is important to note that the Del Forno study did not use indexed LV measurements and included patients with severe degenerative MR and LV dilatation [24]. Thus, they looked at a slightly different patient group with more severe pathology and lacked structured follow-up, which is present in our study.

Similarly, a single centre observational study by van Wijngaarden et al. [7], analysed 125 patients, finding a significant reduction in ILVESV in the *Respect* group (*p* = 0.043) but not the *Resect* group when comparing from baseline to one year postoperatively [7] The observational nature and limited geographical distribution of the Wijngaarden study has the potential for bias, undermining its validity and generalisability to other populations as compared to our findings [7, 23]. Furthermore, unlike our study, the limited group size and lack of data between discharge (5 days; IQR 4–6 days) and one year follow up may limit Hibino et al. and van Wijngaarden et al.’s ability to sufficiently identify group differences and track the progression of LV reverse remodelling during the first year of recovery post-MVr [7, 23].

In the early postoperative period, at 12 weeks, we found no significant difference between Resect and Respect techniques in ILVESD, ILVESV, ILVEDV and ILVEDD. According to the current literature, Respect techniques allow for a more ‘physiological repair’ through preservation of the valve geometry and leaflet mobility. Previous studies by Imasaka et al. (2015) and Mazine et al. (2018) have reported improved postoperative LV efficiency in patients undergoing *Respect* techniques due to the technique’s maintenance of the ventriculo-annular continuity [28, 29]. Despite the borderline difference in ILVEDV at 12 weeks (*p* = 0.06) between *Resect* and *Respect* groups, the difference from baseline to 12 weeks (*p* = 0.60) and 52 weeks (*p* = 0.58) was not significant. This suggests that the MVr technique used did not have clinically or statistically significant impact on the overall recovery of the LV.

Another key finding from this sub-analysis is the confirmation of a two-stage phased process model for LV reverse remodelling progression post-MVr, but the phases did not align with Le Tourneau’s model [8]. Although we found a significant decrease in ILVEDV (*p* < 0.001) in the first stage, we observed an increase in ILVESV (*p* = 0.03). This was contrary to the model, which expected the ILVESV to stay the same during the first phase. In the second phase, we observed a significant decrease from baseline in both ILVESV and ILVEDV, in line with the proposed model [8].

Our study’s ability to compare *Resect* and *Respect* techniques and demonstrate the early and late changes in LV end-systolic/end-diastolic volumes and diameters post-MVr provides further clarity, as compared to previous studies [7, 23, 24].

This study has several strengths. Firstly, we have used high quality data from a large-scale multicentre RCT. Secondly, highly skilled cardiac surgeons, who met the trial’s required case experience criteria performed all the MVr operations in this trial. This ensured that there was similar relative expertise of the surgeons and effectively controlled for variability due to the learning curve. Furthermore, the decision to keep the early postoperative period at 12 weeks has ensured our study provides a better insight into the progression of LV reverse remodelling from index surgery to one-year post-MVr. Finally, all echocardiographic data was reported by a core laboratory, reducing risk of reporting bias.

### Limitations

This study is likely to be underpowered, particularly given the discrepancy between group size, hence these results should be deemed as hypothesis generating only. Secondly, this was an observational study and is at risk of bias by confounding factors. For example, we found there to be a significant difference between ring sizes between *Resect* and *Respect* groups, with the *Resect* group having a larger ring size than the latter (31 vs. 33), which was unexpected and could not be theoretically or practically explained. Thirdly, the use of three-dimensional strain echocardiography measurements, as in the van Wijngaarden study, may have provided a more holistic picture of LV changes and function [7]. Finally, we recommend a longer follow-up period in future studies to assess the long-term impact of *Resect* and *Respect* approaches on MR and indexed LV diameters and volumes over time.

## Conclusion

This study shows that in patients with MR, the MVr technique, *Resect* versus *Respect*, has no significant impact on LV reverse remodelling progression at one year postoperatively. Both techniques led to similar significant improvements in ILVESD, ILVEDD, ILVEDV and ILVESV after MVr at 52 weeks.

## Data Availability

Data will be shared on request to the corresponding author.
